# Supplementary material for: Anti-Trypanosomatidae Activity of Essential Oils and Their Main Components from Selected Medicinal Plants
Source: Molecules. 2023 Feb 2;28(3):1467. doi: 10.3390/molecules28031467 (PMC9920086; doi:10.3390/molecules28031467)
Supplement: Supplementary file 1 [file molecules-28-01467-s001.zip › molecules-2195370-supplementary.pdf]

Supplementary Material

Anti-Trypanosomatidae Activity of Essential Oils and Their Main Components from Selected Medicinal Plants

María Bailén [1,*], Cristina Illescas [1], Mónica Quijada [1], Rafael Alberto Martínez-Díaz [1,†], Eneko Ochoa [2], María Teresa Gómez-Muñoz [3], Juliana Navarro-Rocha [4] and Azucena González-Coloma [5]

[1] Department of Preventive Medicine, Public Health and Microbiology, Faculty of Medicine, Universidad Autónoma de Madrid, 28049 Madrid, Spain
[2] Research and Development Division, AleoVitro, 48160 Derio, Spain
[3] Department of Animal Health, Faculty of Veterinary Sciences, University Complutense of Madrid, 28040 Madrid, Spain
[4] Centro de Investigación y Tecnología Agroalimentaria de Aragón, Unidad de Recursos Forestales, 50059 Zaragoza, Spain
[5] Instituto de Ciencias Agrarias, CSIC, 28006 Madrid, Spain
* Correspondence: maria.bailen@uam.es
† Deceased.

Table S1. Selected plant species, geographic location, coordinates, voucher numbers and extraction method.

Family	Species	Localization and coordinates	Voucher number	Extraction method
Asteraceae	*Tanacetum vulgare*	Ejea de los Caballeros (Spain) (42.13460057158429, -1.209717966420778)	R310457	HD
Lamiaceae	*Lavandula x intermedia "Grosso"**	Ejea de los Caballeros (Spain) (42.13460057158429, -1.209717966420778)	R310458	SD
	*Lavandula x intermedia "Grosso"**	Ejea de los Caballeros (Spain) (42.13460057158429, -1.209717966420778)	R310458	HD
	Salvia blancoana	Ejea de los Caballeros (Spain) (42.13460057158429, -1.209717966420778)	R310459	HD
	Thymus mastichina	Ejea de los Caballeros (Spain) (42.13460057158429, -1.209717966420778)	R310460	HD

*Sterile clones multiplied by cuts.

Table S2. Chemical composition of highly active species EOs: *L. luisieri* (1 and 2) (Ll1 and Ll2), *M. suaveolens* (Ms); *S. hybrid* (Sh), *S. montana* (Sm), *T. vulgaris* (Tv), and *T. zygis* (Tz). RI, Retention index; RT, Retention time; HD, EOs obtained by hydrodistillation; SD, EOs obtained by steam distillation.

Compounds	RI	RT	Ll1		Ll2		Ms		Sh	Sm		Tv		Tz	
			HD	SD	HD	SD	HD	SD	HD	HD	SD	HD	SD	HD	SD
α-pinene	937	3.81	5.70	4.90	2.84	2.56	1.06	0.94	4.34	0.94	0.36	1.07	1.44	1.71	0.43
camphene	950	4.02	0.63	1.07					6.57	0.47	0.17	0.50	1.14	2.09	0.48
sabinene	974	4.34							0.72						
β-pinene	982	4.40	0.16				1.80	1.65	10.92	0.37	0.23	0.45	0.54	0.38	0.18
α-terpinene	1020	5.00							0.05	2.59	1.90	1.44	1.03	1.55	0.46
p-cymene	1026	5.15	0.67	0.63					2.42	18.47	11.75	22.26	31.66	18.34	8.04
limonene	1029	5.18	1.06	0.99			5.60	6.63						1.15	0.53
1.8-cineole	1034	5.24	4.54	1.08	0.90	1.97	1.34	0.04	20.59	0.86	0.85	2.34	2.26		1.51
ocimene	1043	5.48		0.51					0.08						0.36
γ-terpinene	1060	5.72							0.41	11.72	12.36	5.74	2.76	8.95	1.88
D-fenchone	1089	6.31	20.03	1.33	2.50	1.33									
linalool	1102	6.45	4.17	0.27	1.08	2.16	1.21		0.38	1.31	1.09	3.78	3.48	5.40	11.61
camphor	1148	7.44	13.37	34.96	49.20	4.55	0.33		14.23	0.12	0.11	0.53	0.87	1.02	2.68
borneol	1160	7.80			1.25	1.58	0.60	0.39		1.96	1.31	1.03	2.03	6.26	2.50
trans-bornyl acetate	1165	7.88							12.87						
4-terpinenol	1181	8.19	0.74					0.25	0.69	1.46	0.94	1.53	0.76	0.75	1.42
linalyl acetate	1257	9.70											0.14		17.92
trans-α-necrodyl acetate	1283	10.47	12.39	18.77	13.15	18.06									
lavandulyl acetate	1285	10.53	5.55	6.15											
lavandulol	1286	10.54			5.91	7.89									
thymol	1287	10.58								17.29	7.08	43.77	28.15	39.45	21.17
carvacrol	1299	10.84								32.59	41.32	2.42	2.32	5.01	1.36
piperitenone	1342	11.77					53.39	20.77							
piperitenone oxide	1365	12.28					22.83	36.97							
β -caryophyllene	1421	13.55	0.41	0.61	0.12	3.65	1.43	6.07	3.90	2.12	6.07	2.43	5.77	1.42	4.00
germacrene D	1484	14.87	0.37	0.62		7.48	0.82	7.39			0.31	0.07	0.14		1.33
α -bisabolol	1684	18.94													5.45

<5%	5-10%	10-15%	>15%

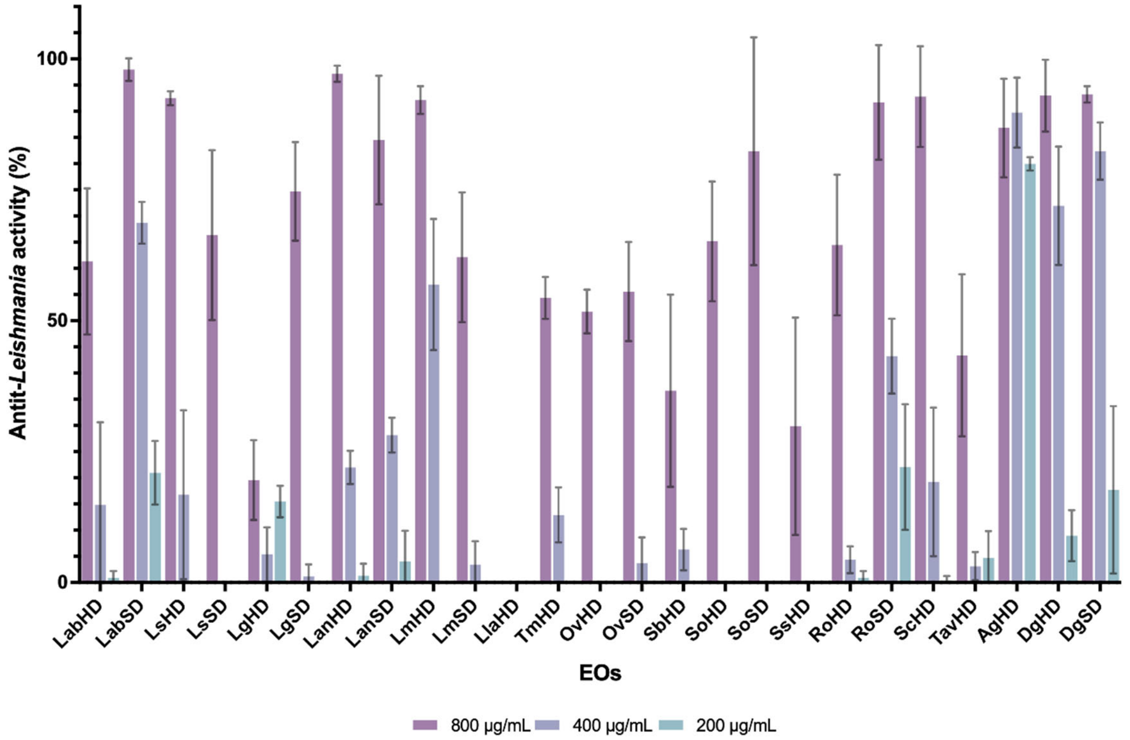

(A)

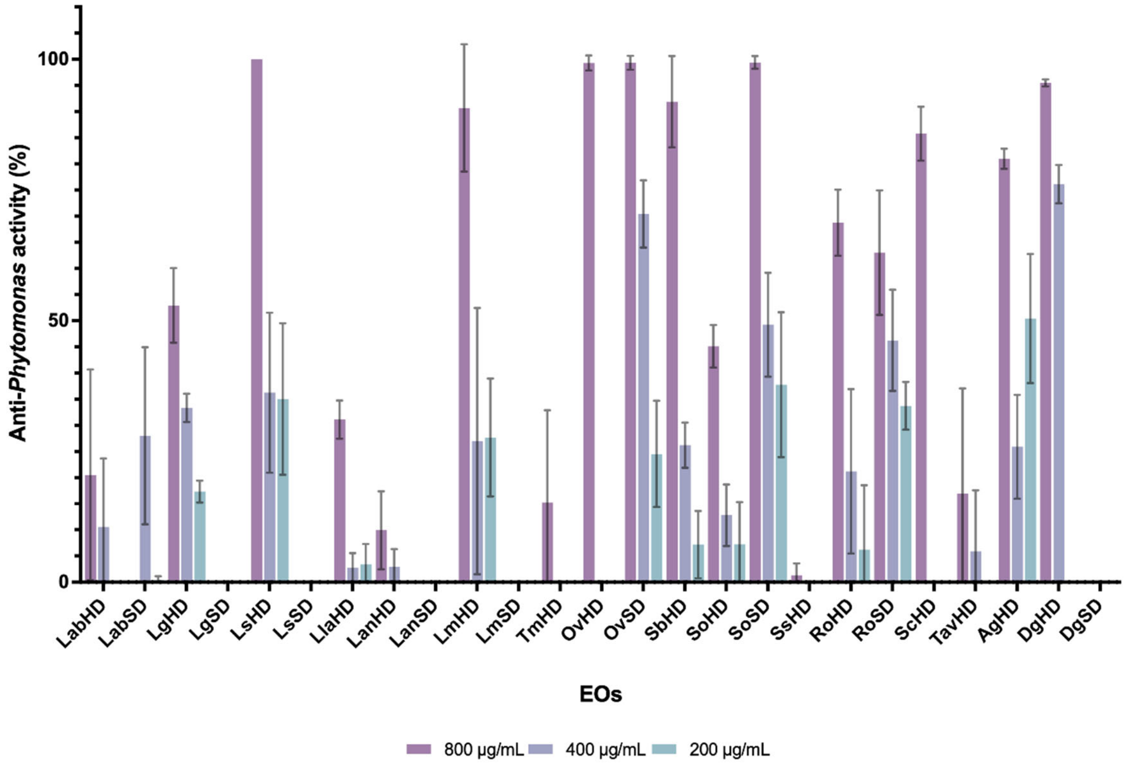

(B)

Figure S1. Percentage of antiprotozoal activity of EOs obtained by hydrodistillation (HD) and steam distillation (SD) from *L. x intermedia* "Abrial" (Lab), *L. x intermedia* "Grosso" (Lg), *L. x intermedia* "Super" (Lsu), *L. lanata* (Ll), *L. angustifolia* (La), *L. mallete* (Lm), *T. mastichina* (Tm), *O. virens* (Ov), *S. blancoana* (Sb), *S. officinalis* (So), *S. sclarea* (Ss), *R. officinalis* (Ro), *S. chamaecyparissus* (Sc), *T. vulgare* (Tav), *D. graveolens* (Dg). A. Anti-*Leishmania* activity. B. Anti-*Phytomonas* activity of EOs.

Ll1 HD

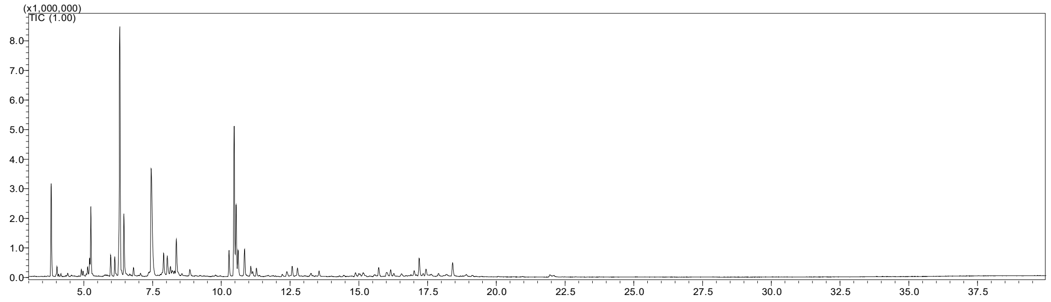

Ll1 SD

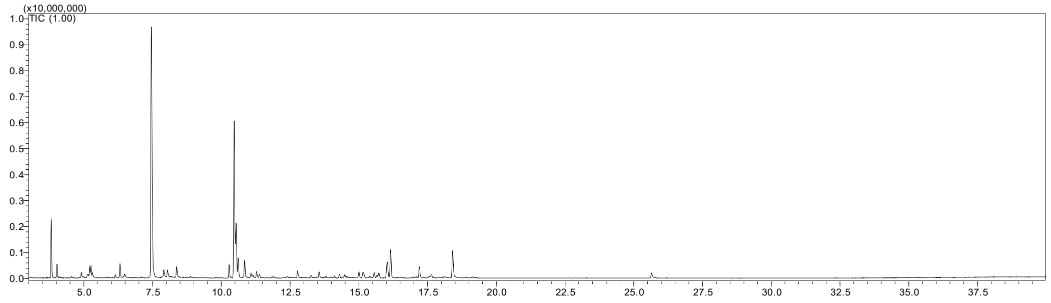

Ll2 HD

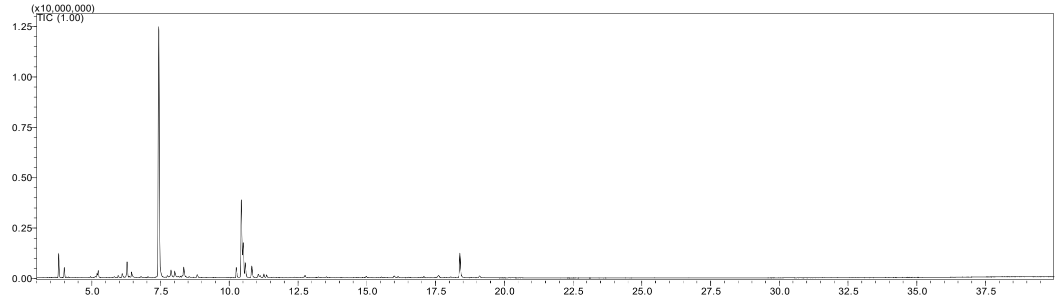

Ll2 SD

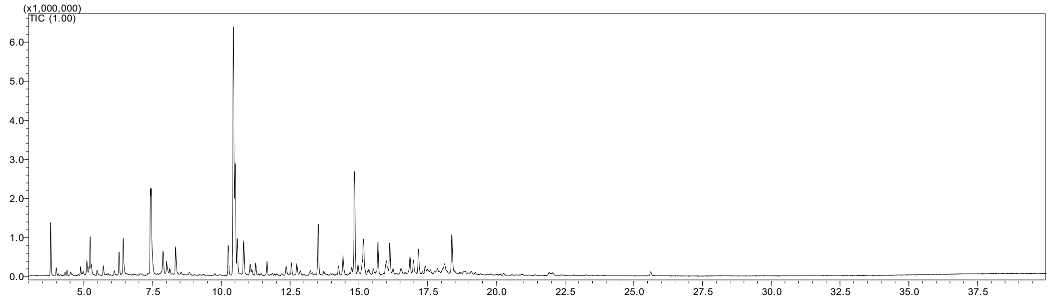

Ms HD

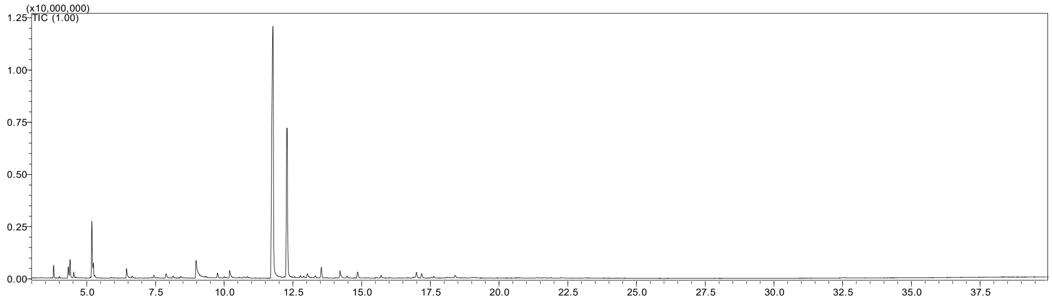

Ms SD

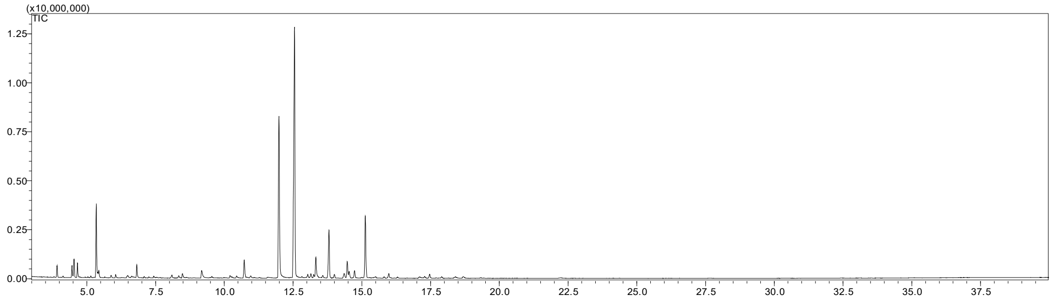

Sh HD

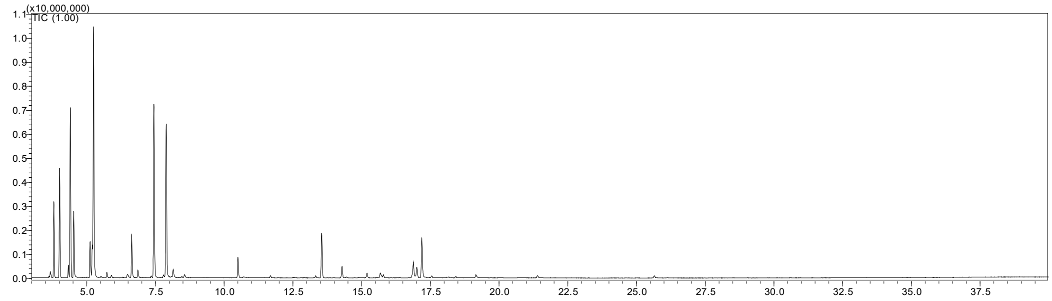

Sm HD

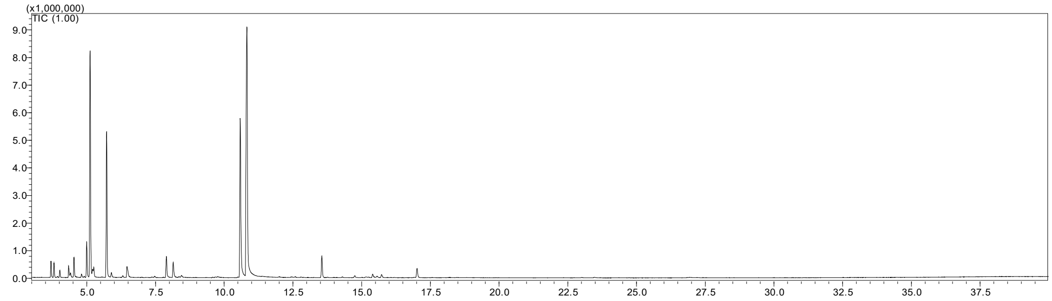

Sm SD

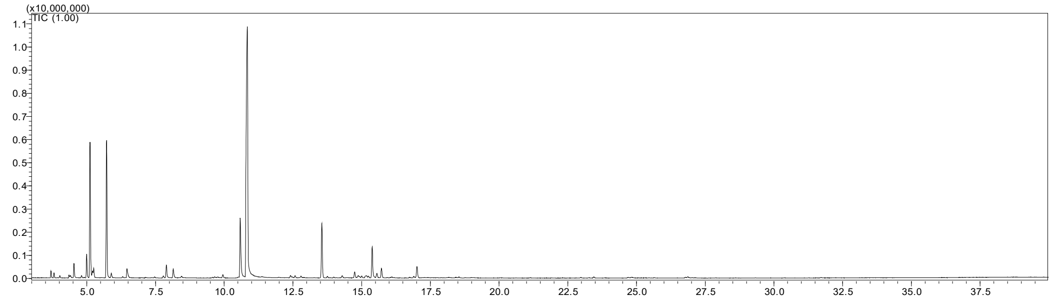

Tv HD

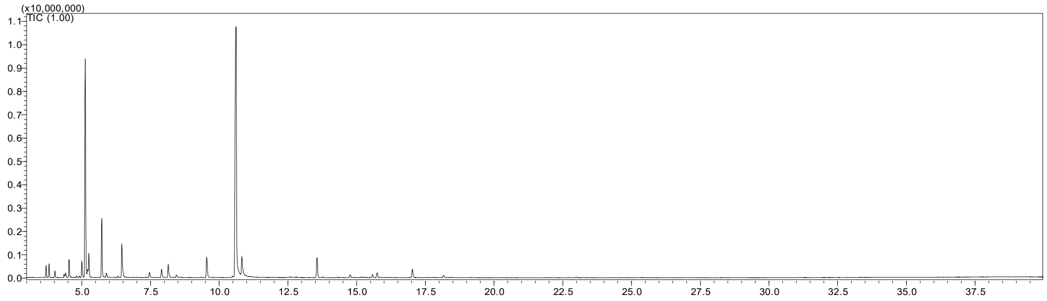

Tv SD

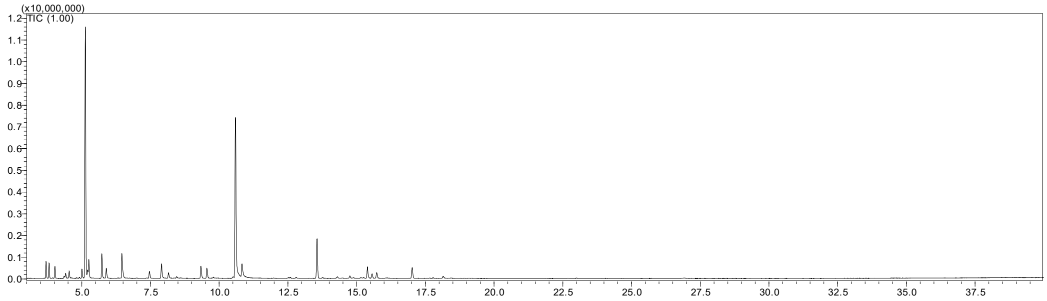

Tz HD

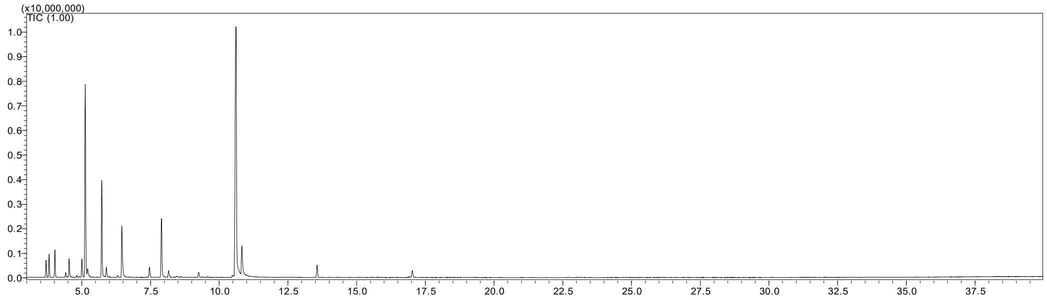

Tz SD

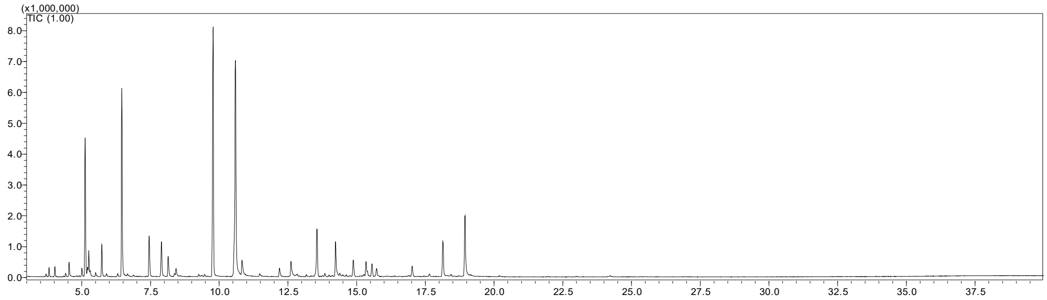

Figure S2. Chromatograms of the most active species EOs: *L. luisieri* (1 and 2) (Ll1 and Ll2),*M. suaveolens* (Ms); *S. hybrid* (Sh), *S. montana* (Sm), *T. vulgaris* (Tv), and *T. zygis* (Tz); HD, EOs obtained by hydrodistillation; SD, EOs obtained by steam distillation.
